# Materia Medica and New Remedies

**Published:** 1877-12

**Authors:** Thomas D. Washburn


BUFFALO

### Medical and Surgical Journal.

VOL. XVII. DECEMBER, 1877. No. 5.

### Original Communications.

———:o:———

ART. I.—*Materia Medica and New Remedies.* By THOMAS D. WASHBURN, M. D.

Read before the District Medical Society of Southern Central Illinois.

In discussing the question of Materia Medica and New Remedies, we propose to limit our remarks to a single generation, while we shall confine ourselves mostly to the results which have been garnered in the last decade.

In taking a general survey of the forces brought to bear in the treatment of disease, the most prominent factor will be found in our better knowledge of hygiene, and the daily use intelligent practitioners make of its laws and principles in controlling, eradicating and preventing it.

The vast improvement in chemical analysis, the wonderful advancement in physiology, and the accumulated facts and products given us by a protracted civil war, as well as the grand numerical results in hospital practice; the use of the microscope, vivisection and anæsthetics; the demonstration of the cell theory, and the unraveling of the intricate nerve fibers and ganglia by the special anatomist; these combined have had a mighty influence on therapeutics. Nor should we forget to notice the rise of *positivism*, the unwillingness to accept anything but facts or demonstrations, which the scientists and students of nature demand, and which alone, they will recognize. The effect has been a rapid winnowing of truth, a rigid exclusion of rubbish, a general sloughing of shams, calculated to astound some of our fossilised and moss-covered brethren.

The knowledge of Materia Medica and therapeutics is second only to the knowledge requisite to *diagnose* correctly. In your intercourse with physicians you have often been surprised at the apparent ease and accuracy with which some arrive at the condition, the morbid derangement in the case before them. They seem to arrive at just conclusions by mere intuition and seldom make mistakes; but when called to prescribe, many of them exhibited the most barren therapeutics, a perfect maze and muddle of materia medica. They have no acquisitions in this direction; calomel, quinine, arsenic, iron, dovers powders and morphine cover the sum total of many whose name is supplimented with an M. D. I have been amazed at the narrow limit of their prescriptive power. We are all subject to more or less criticism for our attachment to *pets*, to our routine medication. You detect it in the most eminent prescribers. We are slow in giving up old and tried friends for untried and doubtful ones, and it is right, but when progress is written all over every thing pertaining to medicine, we have no business to ignore and neglect the generous aid which is extended us by the intelligent pharmaceutist, our colaborer and special friend. Worth like water is said to find its level, and medicines of genuine utility observe the same line; true, now and then an enthusiast will put a good remedy *hors de combat* by his fulsome flatteries and foolish laudation, and true worth remain overlooked by its modest pretensions and unheralded virtues, or become supplanted by some *foreign* rival; but in this day of broad enlightenment, when chemistry stands ready to analyse a sunbeam, and science with its thousand votaries grasps eagerly at every manifestation of nature, whether in the animal, vegetable or mineral kingdom, and a vast field of observation is opening rapidly to the energy of civilization, both on the Pacific slope,

Central and South America, the almost unknown regions of China and middle Asia, swelling our already plethoric Materia Medica; while physiology and pharmacy are alive to utilise every thing of therapeutic value; surely we cannot afford to shut our eyes and stand indifferent to the lavish wealth which surrounds us. The chains of prejudice and the horror of innovation should not paralyse our manliness and crush out our professional freedom.

Do not misunderstand me, I do not advocate a reckless use of *new* remedies. The word *new* has no charms for me, further than it is associated with worth and intrinsic value. The intelligent Pharmacist, the crucial test of chemical experience, which our numerous hospitals and dispensaries afford, with the natural history and generic association of the plant or other element used, all confirm or reject at once the value or worthlessness of any new candidate for professional favor or recognition. Look at the verdict in cundurango after a fair and impartial trial, convicted, sentenced and hung higher than Haman, and deader than an Insurance Company or a Savings Bank; of course ordinary caution and discrimination are to be exercised in making your selection of medical agencies. The member from Sangamon, who reported on this subject at the last meeting in Chicago, justly remarked, that, "the exhibition of drugs in the treatment of disease is somewhat empirical and necessarily so. It is impossible from the physical appearance, habits and chemical constituents of any plant or mineral substance to say positively its therapeutic value, or classify it as a remedy. Its botanical history and chemical constituents may give the direction, or a hint may be taken as to its value and application, but it remains for the clinic to test and verify the value, and properly place it as a remedy."

We shall better appreciate the advance, which has been made, by glancing a moment at the contents of the country doctor's saddle-bags a generation since; calomel, jalap, rhubarb, ipecac, nitrate potassa, paragoric, laudanum, sweet spirits nitre, gum assafœtida, blister ointment, senna leaves, senega, serpentaria, valerian and pink root, quassia and sulphur; no fluid extracts, no bromides, not even chlorate of potassa, no veratrum, gelseminum or belladonna, few had quinine or morphia, they used the crude bark and opium; no ether or chloroform, no glycerine or carbolic acid and only a moderate acquaintance with iodine; now, in addition to these established favorites, we have cincho-quinine, cinchonidia, the carbolates, hydrastin, podophylin, chloral, nitrate of amyl, cannibis indica, jaborandi, lactic acid, eucalyptus, santonine, salycilic acid, stillingia, physostigma, phosphide of zinc, iodoform, and a score of others of less pronounced efficiency, with any amount of new combinations and semi-officinal preparations knocking at the door for recognition and favor; of these the following have been so far adopted as to receive a place among standard authorities, viz: eucalyptus, hydrastin, stillingia, gelseminum, jaborandi, physostigma, podophylin, santonine, and a formidable list of compounds of iron, iodine, bromine and phosphorus; others have come to the front which had barely a name in the books, as salicin, the product of the willow, highly recommended in rheumatism (acute) also in diarrhœa, and as a general tonic and prophylactic; it seems to combine the *tonic* element with an astringent, and is well borne by the stomach. The phytolacca decundia, (poke-root) has had a popular reputation for years for chronic as well as acute rheumatism; it is applicable to a variety of conditions, obstinate cases of mastitis have been relieved, and neuralgic and rheumatic complications not a few. Dr. Hamill of Chicago, three years ago, read a paper on its use combined with Tinct. Guaiacum in cases of rheumatism, dysmenorrhœa and tonsilitis, the result of several years trial, and very efficient. Dose of the Fld. Ext. and Tr. Guaiac equal parts, a teaspoonful after each meal and bed time. Aposynum cannabium (American Indian Hemp,) supplemented the report on Drugs and Medicines at the last meeting of the State Society in Chicago, and elicited warm commendations from some of our most prominent practitioners, as an article worthy of our confidence in anasarca and dropsical affections doing its work in a very quiet unostentatious way, spiriting away the effusion without apparent violence to any function.

Phosphorus has attracted much attention for some time, both from its chemical and physiological relations; but the mass of the profession have been slow in adopting it on account of the irritation often manifested in its exhibition, and the difficulty of securing a safe and reliable menstruum; more recently the phosphide of zinc has won confidence, and seems likely to become an efficient and trustworthy remedy; in *New Remedies* for July 1875, it is spoken of highly in the treatment of chlorosis, anæmia, and especially in those recovering from uterine hemorrhage with neuralgia. It is best given in pill form, watching the effect and following with some acid tonic. Ergot has probably grown more rapidly in the confidence of the profession, and presents a wider range of application than any known remedy. It possesses great value. Aconite, belladonna, gelseminum and veratrum viride are constantly extending their sphere of usefulness, more especially in the neuroses; the latter has proved itself valuable in hemorrhage, and that most formidable of all diseases, eclampsia.

There is perhaps too much fashion in medicine; a remedy is blown into notoriety by accident, or special advertising, and the world goes crazy after it. Hamamelis is a specimen of successful "*gassing*," you find it in every drug-house, and on almost every toilet; as innocent as water and far more worthless. Many remedies have been supplanted by others, either more agreeable or more efficient and gradually become obsolete, such are sanguinaria, serpentaria, columbo, quassia, spegelia, jalap, scammony, comfrey and a host of lesser lights; others are coming to the front and assuming large pretensions for those so young in years; of such are nitrate of amyl, eucalyptus, jaborandi, and a few of our western cousins, grindelia, robusta and squarrosa, yerba santa, beberis aquifolium; a passing notice of these and I will close. Nitrate of amyl was brought forward more prominently by Wier Mitchell, in 1875, as useful in epilepsy, hysteria and kindred conditions. Bartholow in his materia medica, endorses its use and recommends it in spasmotic asthma, tetanus, hydrophobia and strychnia poisoning. Mrs. Dr. Jacobi speaks highly of it in neuralgic dysmenorrhœa. It is given by inhalation, (three to eight drops.)

Eucalyptus is a powerful diaphoretic, good as a tonic in dyspepsia, flatulence and palpitation, especially useful in vesical catarrh, chronic nephritis, and in convalescence of intermittent and debility from defective assimilation. Dose of Fld. Extract fifteen drops to a drachm. Its anti-periodic power is not well established.

Jaborandi is another new remedy, not officinal, attracting much attention and possessing a power unlike anything previously known. It excites profuse perspiration speedily, and is accompanied with equally profuse salivation. It increases the frequency of the pulse, but the arterial tension is diminished. It is indicated in anasarca, ascites, pleuritic effusions, nephritis and sub-acute rheumatism, it has been found useful in diabetes, tetanus and and fevers, also in bronchial asthma, and as a galactagogue. It is likely to assume much prominence and add greatly to our remedial measures. Dr. Hutchins, president of King's county Medical Society, N. Y., presented an exhaustive paper on its merits last June, and Dr. Bartholow gives it special praise. Dose of the tincture half a drachm to a drachm.

And now, we will introduce our new friends from the Pacific slope; grindelia robusta and grindelia squarrosa seem to be near akin, with very similar therapeutic powers, the former has been before the profession some three years, and has some reputation in the treatment of asthma, chronic pneumonia, chronic bronchitis, iritis, hay-fever and gonorrhœa.

The *Yerba Santa*, which has the imposing title of "eurodictyon culafornicon," has received favorable notice by the American Pharmaceutical Association. "This plant grows thriftily among the rocks in northern Mexico, southern and middle California, and is an evergreen shrub, about four or five feet high; its leaves are leathery but brittle, and present a varnished appearance on the upper side as do also the stems, owing to the presence of a resinous body which pervades the plant." The leafy top is the portion used. The flower is a light purplish blue. A syrup of the resin is made by trituration, which has a delicious fruity odor, and a taste resembling pine apple. The June number of *New Remedies* has a description and plate of the plant. It has been a popular remedy among the natives for years. The Spaniards call it the "ages plant," and chew it, and make a tincture with whisky. Dose one-fourth to a drachm. Dr. Bundy of California, thinks it unequaled in "laryngeal and bronchial troubles." Dr. Newell of St. Louis, says it is an excellent expectorant and diuretic. A patient of mine suffering from bronchial disease and obstinate cough, gathered quite an amount of it, by direction of his physician while in California, and spoke of it in high terms. He presented me with some, which the members can examine. I have found it palliative and useful in hoarseness and sore throat.

Dr. Bundy also speaks highly of the "beberis acquifolium," a flowering shrub cultivated in the California gardens, two to six feet high, with yellow flowers. The root is the part used, which is of a bright golden color, and intensely but pleasant bitter. Its virtues seem to be alterative and tonic, and specially anti-syphilitic. Dose of fluid extract 15 to 30 drops four times per day.

Had I not trenched already on your valuable time, I should have been glad to have drawn your attention to the topic of inhalation as well as the hypodermic use of remedies, but I forbear. They are rapidly being interwoven in our therapeutics, and we cannot afford to have specialists carry off laurels which legitimately belong to us.

Electro-therapeutics is another branch which indolence and ignorance have too long kept out of view. A man should be familiar with every thing pertaining to his profession, and only so far as he gives evidence of such knowledge is he entitled to success and appreciation.

As one anxious to stimulate both the junior and senior members of the profession in the right direction, allow me to make a single prescription. If there is a single branch of the profession or tributary leading to the same, which you have not yet explored; do as I have done, *write an article upon it* and present it to the Society. It will give you an appreciative sense of your ignorance, and possibly save you from some stupid blunder in practice.

